# Combining Stellate ganglion block and intravenous regional anesthesia to treat complex regional pain syndrome: A 19-years’ experience in a tertiary center

**DOI:** 10.15537/smj.2022.43.10.20220318

**Published:** 2022-10

**Authors:** Abdelkarim S. Aloweidi, Sami A. Abu Halaweh, Mahmoud M. Al-Mustafa, Islam M. Massad, Ibraheem Y. Qudaisat, Aws M. Khanfar, Jihad M. Ajlouni, Shaher T. Alhadidi, Ahmad I. El-Share, Mohammad A. Jarbeh, Anas A. Al-Oweidi

**Affiliations:** *From the Department of Anesthesia and Intensive Care (Aloweidi, Abu Halaweh, Al-Mustafa, Massad, Qudaisat, El-Share, Jarbeh); from the Department of Orthopedics (Khanfar, Ajlouni Alhadidi), School of Medicine; and from the Department of Internal Medicine (Al-Oweidi), Faculty of Medicine, University of Jordan, Amman, Jordan.*

**Keywords:** complex regional pain syndrome, causalgia, Pain, regional anesthesia, reflex sympathetic dystrophy, intravenous regional anesthesia, Stellate ganglion block, sympathetic dysfunction

## Abstract

**Objectives:**

To study reduction in pain score after treatment with intravenous regional anesthesia (IVRA) and Stellate ganglion block (SGB) combination on complex regional pain syndrome (CPRS) patients and to quantify patients’ satisfaction with treatment and occurrence of complications.

**Methods:**

This is a record-based retrospective review carried out in 2020, targeting patients treated in the University of Jordan Hospital, Amman, Jordan, over the years 2002-2020.

**Results:**

Among 99 patients, a significant drop in pain scores occurred in 88% of the patients’ sample. Gender, age, type of CRPS, and duration of symptoms didn’t affect statistical results. An average of 8.6 sessions needed to achieve 50% drop in pain score, and 2-3 sessions for first clinical improvement. Patients with previous application of plaster of Paris had increased success rates.

**Conclusion:**

We find it practical, inexpensive, safe, and straightforward to combine SGB with IVRA for CRPS patients.

**S**ince its description in the literature (around 1870) complex regional pain syndrome (CRPS) has proven to be one of the most difficult pain disorders to treat, and it continues to cause major limitations to the daily activities of its victims. Diagnosis of CRPS is basically clinical using the the International Association for the Study of Pain (IASP) and Budapest criteria.^1^ Two types of CRPS exist (type I [without nerve injury], and type II [with documented nerve injury]).^2^ Complex regional pain syndrome is more prevalent among females.^3^

Complex regional pain syndrome is one of the most debilitating pain syndromes causing major psychological consequences, most commonly depression, and approximately 30% of patients have considered suicide during their illness.^3^

Sympathetic dysfunction is a major factor in CRPS pathogenesis, and various approaches to sympathetic blockade have been used for treatment.^4-6^

Lidocaine has been used in intravenous regional anesthesia (IVRA) and Stellate ganglion block (SGB) as an adjunct during surgical repair, with physiotherapy sessions to improve success in treating CRPS.^7-10^ The mechanism of SGB may involve central mechanisms altering nerve growth factors, resulting in relief of symptoms for durations longer than expected from local anesthetic action alone.^11^

The available evidence on CRPS in the Middle East and North Africa is limited.^12^ A survey was carried out on the prevalence of CRPS in the Jordanian population and studied the effectiveness of previous treatments.^13^ In our search in the literature, unlike peri-operative use of IVRA, we found no previous use of steroids in intravenous regional blocks for CRPS.

A practical, easy, and inexpensive method of treating CRPS is greatly needed. In this study, we present our experience with carrying out a combination of a SGB and IVRA for the treatment of CRPS patients in settings with limited resources, and evaluate the effectiveness of that combination in reducing pain scores in our patients.

The objectives of this study included; I) studying reduction in pain score after treatment with IVRA, SGB combination on CPRS patients, and to quantify patient satisfaction with treatment and occurrence of complications; II) studying the effect of age, gender, total number of blocks, plaster of Paris application, and duration of symptoms on treatment success and number of therapeutic sessions needed to achieve success; and III) comparing outcomes of the combination for upper and lower limbs.

## Methods

This is a record-based retrospective observational study carried out in 2020 at the University of Jordan Hospital, Amman, Jordan.

We referred to pain clinic data to search for CRPS patients’ data, over the years 2002-2020, and collected 103 records of patients diagnosed with CRPS, after which we applied inclusion and exclusion criteria to obtain 99 eligible records.

Inclusion criteria included: I) all cases of CRPS patients who were referred for invasive chronic pain management from 2002-2020; II) who had not responded to oral analgesics and physiotherapy in 4-8 weeks; and III) had pain scores of >8 of 10 at the beginning of treatment sessions.

**Figure 1:**
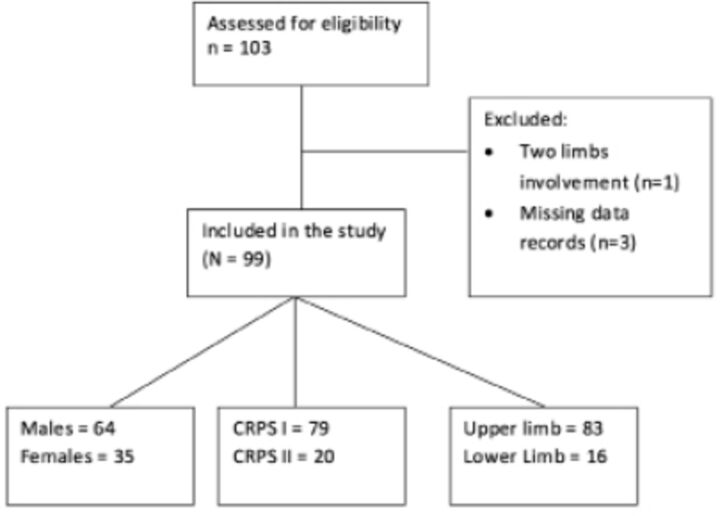
- A STROBE flowchart.

Exclusion criteria included: I) cases of patients with more than one extremity involved simultaneously; II) those who did not undergo combined IVRA and SGB treatment; and III) those whose medical records did not contain complete data (Figure 1).

The diagnosis of CRPS was based on the IASP criteria before 2007, then on the Budapest criteria after 2007.^4^

In each therapeutic course, all patients received a combination of IVRA and SGB ipsilaterally in the same session. All patients were monitored with the standby IV access on the unaffected limb. Intravenous regional anesthesia was lidocaine 0.5-20% mL (Lidocaine-Braun, Germany) for children, and 40 mL for adults (through a 22G cannula after exsanguination of the limb and inflation of the pneumatic tourniquet to 100 mmHg above systolic pressure for 30 minutes). Simultaneously, a SGB was carried out with 5 mL of plain lidocaine 1% on the ipsilateral side. Follow up sessions were carried out weekly.

Data collected included: patients’ age, gender, medical history, type of symptoms, type of CRPS, precipitating factor, duration of symptoms, type of regional anesthesia treatment, total number of courses of regional blocks and the course at which first improvement was encountered, occurrence of complications from treatment, referral to physiotherapy, and history of plaster of Paris application.

We defined a patient’s first improvement as the first course at which the VAS score had dropped to 7 of 10 or less. Success of treatment was defined as a pain score dropping to less than 5 of 10 (a 50% reduction in pain score).^14,15^ Satisfaction with treatment was measured with a 10-point Likert scale ranging from 1-10 (1=meaning least satisfied and 10=meaning most).

This work obtained an approval from the Institutional Review Board (IRB) of Jordan University Hospital, Amman, Jordan, before data collection (Grant No. 10/2020/15019). Due to the retrospective nature of this study, our institutional IRB waived the requirement to obtain written informed consent before starting data collection. We adhered to the tenets of the Declaration of Helsinki and Good Clinical Practice guidelines.

### Statistical analysis

Microsoft Excel 2007 software was used for data entry and data were then migrated to the Statistical Package for the Social Sciences software, version 25.0 (IBM Corp., Armonk, NY, USA). We analyzed data of 99 eligible cases; final sample’s data, features, and characteristics were summarized using mean ± standard deviation (SD) for normally distributed continuous variables and comparison was carried out by the student T-test, the median for non-normally distributed continuous variables. Comparison of means was carried out using Mann-Whitney test and Chi-square analysis for percentages. Significance level was established as *p*<0.050.

## Results

Our patients were in the young age group, with an average of 38.3 years (range: 11-68). Other data is summarized in Table 1.

**Table 1:** - Descriptive summary of general sample data illustrating patients’ improvement (N=99).

Variables	n (%)	Successful treatment	Number of courses for success	Number of sessions for improvement
***Gender***				
Males	64 (64.6)	87.5%	7.6	2.2
Females	35 (35.3)	88.5%	10	3.4
***CRPS***				
Type I	79 (79.8)	88%	8.44	2.66
Type II	20 (20.2)	85%	9.18	2.53
***Affected limb***				
Upper	83 (83.8)	86.7%	8.7	2.4
Lower	16 (16.2)	93.7%	7.9	3.5
***Precipitating factors***				
Trauma	87 (87.9)	-	-	-
Surgery	6 (6.1)	-	-	-
Burn	1 (1.0)	-	-	-
Unknown	5 (5.0)	-	-	-

Values are presented as a number and precentage (%). CRPS: complex regional pain syndrome

Although CRPS is basically a clinical diagnosis, patients were sent for limb X-ray and 3-phase bone scans. Some patients needed electromyography and nerve conduction studies to diagnose nerve injury. Only 12% of the patients had typical 3-phase positivity and 63% had osteopenia on the X-ray. A nerve conduction study was used to diagnose nerve injury (type II CRPS).

Success rate for the whole sample was 87% (defined as a target drop in pain score >50% of baseline). These patients needed an average of 8.59 therapeutic sessions to achieve target. The average number of therapeutic courses needed to reach the first improvement (drop in pain score to less than 7 of 10) was 2.63 sessions.

Males accounted for 65% of the sample, of which 56 (87.5%) had successful treatment, with an average satisfaction score of 5.4 of 10, an average of 7.6 therapeutic courses needed to achieve success, and an average of 2.2 courses to achieve first clinical improvement.

Of the 35 female patients, 88.5% had successful therapy and needed 10 therapeutic courses on average to achieve success, an average of 3.4 therapeutic courses needed to achieve first clinical improvement, and a satisfaction score of 6.1 of 10.

A Mann-Whitney-U test was carried out to study the gender effect on pain scores before and after treatment; it showed no statistically significant difference (*p*=0.56).

Effect of precipitating factors on study outcomes revealed that trauma to the limb was the most common precipitating factor (87 cases) as follows: 45 cases of bone fracture, 34 cases of tendon/soft tissue cutting trauma, and 8 patients with blunt trauma. Six patients developed CRPS after elective surgery for non-trauma causes, one patient after a burn, and 5 cases with no clear cause.

Effect of type of CRPS on the study outcome showed that most patients had type I CRPS (79.8%). Patients with both types of CRPS had an approximate percentage of successful treatment. A Mann-Whitney-U test was carried out to study the effect of the 2 types of CRPS on pain scores before and after treatment; it showed no statistically significant difference (*p*=0.47). The T-test showed no statistically significant difference between the 2 types of CRPS on patient satisfaction (*p*=0.66) and the number of therapeutic courses needed for successful treatment (*p*=0.56). Studying site of affected limb showed that most sample patients (83.8%) had upper limb involvement, with a successful treatment rate of 86.7%, an average of 8.7 therapeutic courses needed to achieve success, an average of 2.4 courses needed to achieve first improvement, and a score of 5.1 of 10 for patient satisfaction with treatment.

Of the 16 patients with lower limb involvement, 93.7% had successful treatment, an average of 7.9 sessions needed to achieve success, an average of 3.5 therapeutic courses needed to achieve first improvement, and a score of 4.8 of 10 for patient satisfaction with treatment.

A Mann-Whitney-U test was carried out to study the effect of affected limb location on pain scores before and after treatment; it showed no statistically significant difference (*p*=0.38).

A total of 68 patients had plaster of Paris cast applied to the affected limb as part of trauma management and 97% of them had successful treatment. A Mann-Whitney-U test did not show a statistically significant effect of Paris plaster application on pain scores before and after treatment (*p*=0.35). There was no statistical correlation between the duration of POP and the number of sessions needed for successful treatment (Spearman’s rho= -0.11).

Only 5 cases had minor hemodynamic changes (3 cases of bradycardia and 2 of hypotension). They were short term, improved with fluid management, and did not lead to serious complications. The most common complication was increased weakness of the affected limb during the procedure (56%), which resolved within one hour after the procedure. This was followed by cuff discomfort (17%), which was resolved in a few minutes after cuff deflation. Only 2 patients had Horner’s syndrome in one of the therapeutic courses with no concomitant hemodynamic changes, and it was completely resolved in a few hours soon after. Other minor complications, such as tinnitus or dizziness, had an incidence of 3%.

Patients with affected lower limbs had lower rates of complications (31%) than patients with upper limb involvement (57%).

In regard to risk factors, our sample had little incidence of chronic illnesses other than (5% with diabetes mellitus, 8% were hypertensive, 2% had ischemic heart disease, 5% had respiratory diseases, 41% were smokers, and none was alcoholic).

As expected from all chronic pain conditions, most CRPS patients face psychological problems, and psychiatrists play an important role in multimodal approaches to treatment, but unfortunately, few of our patients agreed to be referred to a psychiatrist, mostly due to fear of social stigma.^7,8^

## Discussion

Access to chronic pain services in Jordan and the Middle East is limited. This study summarizes our experience (2002-2020) treating patients with CRPS, for whom first lines of therapy had failed, using a combination of IVRA and SGB.

Although the results of our study showed that 88% of our sample had successful treatment (a decrease of >50% in pain score from the baseline). Patients needed 2-3 sessions for first clinical improvement, and 8-10 sessions for pain score to drop below 50% from baseline.

Contrary to the European literature, in which most CRPS patients are elderly and female, our sample patients were young workers and mainly male.^3^ This may be explained by the population in the Middle Eastern region being younger in general, the scarcity of facilities for diagnosing CRPS, limited knowledge of doctors and patients regarding chronic pain conditions, and because in most cases of trauma, patients may have easier access to a chronic pain physician as part of referrals for their continuous care.

The application of Paris plaster cannot be controlled by the chronic pain physician; it is carried out by the primary physician managing the trauma near the time of the incident (before the development of chronic pain). Although its application is known to increase the risk of developing CRPS, in our sample, those with a history of Paris plaster application had a higher rate of successful treatment using the IVRA and SGB combination therapy.^14^

Similar outcomes of a therapeutic SGB in the upper and lower limb groups point to the central mechanism, leading to improved outcomes in the lower limb group as well as in the upper limb group.^11^

The combination of IVRA and SGB appears to be safe, as most complications were minor and self-limiting, and did not hinder the use of this technique for the treatment of CRPS cases.

In general, Jordanians, like other Middle Eastern healthcare providers, have limited knowledge of chronic pain conditions and limited facilities to diagnose and treat CRPS in particular, which led to limited access of patients to chronic pain physicians, misdiagnosis of CRPS by non-specialized healthcare providers, and delayed presentation of many patients to specialized pain services.^11^ Approximately 14% of the sample patients presented to us with symptoms persisting for more than one year (10 patients endured symptoms for more than 3 years, and one was referred after 10 years). Although we did not find a statistically significant effect of the duration of symptoms on short-term outcomes in our sample data (such as a bias due to the small sample size), we believe that earlier treatment enhances better outcomes and earlier return to function.

In contrast to previous research, we found similar results for types I and II CRPS in response to therapeutic sessions in our sample, which may be related to a misdiagnosis of nerve injury or to an occurrence of statistical type B error due to the small number of type II patients.

Regarding cost, it was difficult to obtain actual prices of materials and equipment, but in a rough estimate of our technique, there was no expensive equipment or medications, no need for hospital admissions (it is an outpatient procedure), and patients with low to average income could afford our treatment sessions.

### Study limitations

I) Absence of a control group and non-blinding of data due to the retrospective nature of this study; II) lack of long-term patient follow-up because most patients were referred from other medical centers in distant areas; and III) the small sample size.

Implications for further research includes: a multi-centric randomized controlled trial involving IVRA and SGB for CRPS patients and use of pharmacological additives to this technique.

In conclusion, we found that the combination of IVRA with SGB for the treatment of CRPS patients is practical, inexpensive, safe, and straightforward, with a high success rate for both upper and lower extremity involvement, in both types of CRPS, and equally in both genders and among different age groups.
